# Copper Tolerance Mechanism of the Novel Marine Multi-Stress Tolerant Yeast *Meyerozyma guilliermondii* GXDK6 as Revealed by Integrated Omics Analysis

**DOI:** 10.3389/fmicb.2021.771878

**Published:** 2021-11-18

**Authors:** Ru Bu, Bing Yan, Huijie Sun, Mengcheng Zhou, Huashan Bai, Xinghua Cai, Xueyan Mo, Guijiao Su, Chengjian Jiang

**Affiliations:** ^1^State Key Laboratory for Conservation and Utilization of Subtropical Agro-bioresources, Guangxi Research Center for Microbial and Enzyme Engineering Technology, College of Life Science and Technology, Guangxi University, Nanning, China; ^2^Guangxi Key Lab of Mangrove Conservation and Utilization, Guangxi Mangrove Research Center, Guangxi Academy of Sciences, Beihai, China; ^3^Guangxi Flyment Biotechnology Co. Ltd., Nanning, China

**Keywords:** *M. guilliermondii*, copper tolerance mechanism, antioxidant enzyme genes, integrated OMICs analysis, non-enzymatic antioxidant genes

## Abstract

Various agricultural products used in food fermentation are polluted by heavy metals, especially copper, which seriously endangers human health. Methods to remove copper with microbial strategies have gained interests. A novel *Meyerozyma guilliermondii* GXDK6 could survive independently under high stress of copper (1400 ppm). The copper tolerance mechanism of GXDK6 was revealed by integrated omics in this work. Whole-genome analysis showed that nine genes (i.e., *CCC2*, *CTR3*, *FRE2*, *GGT*, *GST*, *CAT*, *SOD2*, *PXMP4*, and *HSP82*) were related to GXDK6 copper tolerance. Copper stress elevated glutathione metabolism-related gene expression, glutathione content, and glutathione sulfur transferase activity, suggesting enhanced copper conjugation and detoxification in cells. The inhibited copper uptake by Ctr3 and enhanced copper efflux by Ccc2 contributed to the decrease in intracellular copper concentration. The improved expression of antioxidant enzyme genes (*PXMP4*, *SOD2*, and *CAT*), accompanied by the enhanced activities of antioxidant enzymes (peroxidase, superoxide dismutase, and catalase), decreased copper-induced reactive oxygen species production, protein carbonylation, lipid peroxidation, and cell death. The metabolite D-mannose against harsh stress conditions was beneficial to improving copper tolerance. This study contributed to understanding the copper tolerance mechanism of *M. guilliermondii* and its application in removing copper during fermentation.

## Introduction

Copper is an essential trace element for living cells (Bao et al., 2020). Adults need to consume 1.2 mg copper daily for life activities (de Luna et al., 2020), and insufficient copper intake may cause anemia, gastrointestinal discomfort, and eye diseases (Zhang et al., 2019). Copper can bind to proteins, and unbound copper causes protein inactivation, thereby damaging the liver and kidneys and causing various diseases (Que et al., 2008). Copper is also involved in the processes of essential biochemicals, such as superoxide dismutase (SOD) and tyrosinase, as an enzyme cofactor (Viguier and Hulme, 2006; Schwartz et al., 2013). With human interventions and industrial activities, copper discharged from metal mines, smelters, and urban wastewater enters the aquatic system, explaining its increasing concentration in freshwater (Lahman et al., 2015). At present, the soil concentration of heavy metals in China is higher than in those in Europe and the United States but lower than those in India and Africa. This phenomenon may be due to the different levels of industrialization in different countries. The copper content in China is higher than the global average (Yuan et al., 2021). On April 17, 2014, the first batch of national soil pollution survey results showed that one-fifth of the agricultural land was polluted by heavy metals. Heavy metal pollution is severe in central and southwestern China (Wan et al., 2018). Surveys conducted by the Ministry of Land and Resources, the Ministry of Environmental Protection, and the Ministry of Agriculture showed that the total proportion of land with soil pollution exceeding China’s soil heavy metal standards 3 reached 16.1% (Delang, 2017). In addition, 64.8% of China’s 1.4 million hectares of sewage irrigated farmland are polluted by heavy metals, and 12 million tons of grain are polluted by heavy metals every year (Li X. et al., 2018). Among them, the heavy metal pollution in Hunan is the most serious. The copper content reaches 13.85–111.60 mg/kg, which is 1.53 times higher than the background value. In Changsha, eastern Hunan Province, areas such as Zhuzhou, Xiangtan, and Chenzhou have high copper contents (Li X. et al., 2018). Heavy metal pollution has brought huge economic losses to our country. Some studies have taken Beijing as an example to estimate the economic losses caused by pollution. The global loss in the upper reaches of the river is estimated to be 466.63 dollars per hectare, whereas the global loss in the lower reaches is estimated to be 1,133.52 dollars/Ha (He et al., 2014). Excess copper cannot be metabolized and flows to the ecosystem, causing huge economic losses and copper accumulation in the food chain; as a result, many agricultural products are contaminated with copper. Thus, food safety and human health have become a major issue worldwide (McLaughlin et al., 2000; Ling et al., 2007).

Aquatic organisms can also accumulate large amounts of heavy metals, hindering the further utilization of fishery and aquatic product processing wastes containing rich proteins, amino acids, and fatty acids (Blanco et al., 2015). Bartolome et al. (2010) reported that the concentration of copper ions in mussels from the Cantabrian Coast is the highest among heavy metals, reaching 10 and 25 mg/kg dry weight. Traditional methods, such as chemical precipitation, electrochemical methods, membrane separation, and ion exchange technology, cannot effectively remove heavy metals from food because of their technical bottleneck and economic limitations (Wang and Chen, 2009; Nguyen et al., 2013). Currently, excess copper has a negative impact on the fermentation industry. In microbial anaerobic fermentation, copper nanoparticles can cause oxidative damage to cell membranes, inhibit the growth and metabolism of *Moorella thermoacetica*, significantly reduce glucose consumption, and decrease the level of pyruvate metabolism (Wu et al., 2021). Exposure to 600 ppm copper ions for 24 h can alter the glucose fermentation pathway of *Klebsiella* and inhibit the acetic acid metabolism of *Saccharofermentans acetigenes* (Hu et al., 2021; Zhang et al., 2021). Bioaccumulation via microorganisms is a potential technology to remove copper in food materials. Copper can be removed in two steps: cell surface bioaccumulation of cell walls and intracellular bioaccumulation of heavy metal transporters (Duncan and Brady, 1994; Li et al., 2014). Yeasts exhibit good copper bioaccumulation ability (Radic et al., 2017). Microbes have evolved different strategies to resist the toxicity of heavy metals. In bacteria, heavy metals are excreted from the body through efflux pumps (Nies, 1999). For *Saccharomyces cerevisiae*, the transport and absorption of copper are involved in the expression of extracellular copper reductase protein complex Fre1p/Fre2p, the reduction of siderophore-bound iron and oxidization of copper prior to uptake by transporters, and the reduction of copper ions to monovalent copper ion for uptake (Hassett and Kosman, 1995; Georgatsou et al., 1997). Yeast also contains metallothionein-like proteins Cup1 and Crs5, which can sequester excess copper and reduce cell toxicity (Karin et al., 1984; Culotta et al., 1994).

However, heavy metals removed via microbial strategy is limited by environmental factors, such as high salinity, low pH, and various heavy metals; these complex environment factors can inhibit microbial growth and affect the final removal efficiency (Li C. et al., 2016). Therefore, microorganisms with multiple stress tolerance and good heavy metal removal have gained interests. A previous study identified the multi stress tolerant yeast *Meyerozyma guilliermondii* GXDK6 with aroma-producing properties from subtropical marine mangrove wetland microorganisms (Mo et al., 2021). This species can survive 1400 ppm (22 mM) copper ions, which is the highest tolerance concentration reported to date. The copper tolerance concentrations of *Sporosarcina pasteurii* and *Staphylococcus equorum* are approximately 1.5 and 0.8 mM (Sepúlveda et al., 2021). Different researchers have reported that the copper tolerance range of *S. pasteurii* is 0.2–0.5 mM (Kloos and George, 1991), *Bacillus subtilis* 0.2–0.5 mM (Radford et al., 2003), *Streptomyces* sp. 0.5–0.8 mM (Majzlik et al., 2011), *Sphingomonas paucimobilis* 3.9–4.7 mM (Altimira et al., 2012), and *Pantoea* sp. 4.7 mM (Singh and Jha, 2018). Compared with other strains, *M. guilliermondii* GXDK6 has stronger copper tolerance. However, to our knowledge, few studies reported the copper tolerance of *M. guilliermondii*, which remarkably limits the development of *M. guilliermondii* fermentation applications.

Given its remarkable copper-tolerant survivability, GXDK6 is hypothesized to survive under high-copper stress by regulating genes related to glutathione metabolism, antioxidant enzymes, and copper transport to control the expression of copper-tolerant key proteins, which are beneficial to the regulation of cell function and/or production of secondary metabolites. In addition, copper stress induces GXDK6 to regulate carbohydrate metabolism, amino acid metabolism, and secondary metabolite biosynthesis for GXDK6 survival in high-copper conditions. In the present study, integrative omics strategies are performed to investigate the survival mechanism of *M. guilliermondii* GXDK6 under copper stress. This work contributes to understanding the metabolic regulation and to developing important functional metabolites from *M. guilliermondii* GXDK6.

## Materials and Methods

### Strains and Culture Conditions

The *M. guilliermondii* GXDK6 in this study had been deposited in China General Microbiological Culture Collection Center (CGMCC) with CGMCC No. 16007. Yeast cells were grown in Yeast Extract Peptone Dextrose Medium (YPD) containing 2% glucose, 2% peptone, and 1% yeast extracts sterilized at 115°C for 15 min. *M. guilliermondii* GXDK6 living cells were cultured for 12 h at 30°C under aerobic conditions and shaken at 200 r/min.

### Materials

Chromatographically pure yeast extract, tryptone, dextrose, and CuCl_2_⋅2H_2_O were purchased from Thermo Fisher (Corporate Contacts, United States). Chromatographically pure methoxyamine hydrochloride–pyridine and trifluoroacetamide were purchased from Sangon Biotech (Shanghai, China) and used in GC-MS. RNA rapid extraction kit, cDNA first-strand rapid synthesis kit, and real-time fluorescent quantitative polymerase chain reaction (PCR) kit were purchased from Sigma-Aldrich, Inc. (Darmstadt, Germany). Malondialdehyde (MDA) content detection kit, SOD activity detection kit, peroxidase (POD) activity detection kit, catalase (CAT) activity detection kit, total antioxidant capacity detection kit, and primers were purchased from Sigma–Aldrich, Inc. (Darmstadt, Germany).

### Determination of Copper Tolerance

The stock copper ions solution (100 g/L) was prepared by dissolving CuCl_2_⋅2H_2_O in ultrapure water. The GXDK6 seed solution was inoculated into YPD medium containing different copper ion concentrations (0, 200, 400, 600, 800, 1000, 1200, 1400, and 1500 ppm) with 2% (*v/v*), incubated for 102 h at 30°C under aerobic conditions, shaken at 200 r/min, and then sampled. OD_600_ values were measured every 12 h to determine copper tolerance.

The growth curve of GXDK6 under copper stress was established to determine the effect of copper ions on the growth characteristics of GXDK6. The GXDK6 seed liquid was inoculated into YPD medium with copper ion concentrations of 0, 200, 400, 600, 800, and 1000 ppm, incubated for 40 h at 30°C under aerobic conditions, shaken at 200 r/min, and then sampled at specific intervals. OD_600_ values were then measured.

### Morphological Observation of GXDK6 Under Copper Stress

The fermentation broth (1 mL) cultured for 12 h under copper ion concentrations of 0, 600, and 1000 ppm was centrifuged at 10,000 × *g* for 5 min. The supernatant was discarded, and the pellet was collected and washed with ultrapure water five times. An appropriate amount of sample was collected for smear. The morphology of GXDK6 on the conductive glue was observed using scanning electron microscopy (FEI Quattro S, Thermo, United States, made in Czechia). The scanning voltage was set to 10 kV, and the magnification was set to 10,000 times.

### Determination of Copper Ions Removal Efficiency

GXDK6 was inoculated in YPD medium containing 50, 100, 200, 400, 600, 800, and 1000 ppm copper ions and then incubated for 96 h. The sample (2 mL) was collected and centrifuged at 10,000 × g for 5 min. Supernatants were diluted 100 times with ultrapure water. YPD media containing 600 and 1000 ppm copper ions without GXDK6 were used as control. The concentration of copper ions in the diluted fermentation broth without other treatment was measured using inductively coupled plasma emission spectrometry (ICP-5000, Focused Photonics, China) to determine the adsorption efficiency of GXDK6 to copper ions.

### Metabolomics Analysis of GXDK6 Under Copper Stress

Samples were cultured for 12, 24, and 48 h under the conditions of 0, 600, and 1000 ppm copper ions, respectively, and then centrifuged at 10,000 × *g* for 5 min. The supernatant was filtered using a 0.22 μm sterile filter membrane, concentrated through refrigerated centrifugation (Shanghai, China), and then freeze-dried into powder. The powdered samples were derivatized with 0.1 mg/mL methoxyamine hydrochloride–pyridine solution (reagent for GC) for 120 min and then alkylated by trifluoroacetamide (reagent for GC) for 120 min. After completion, the samples were centrifuged at 10,000 × *g* for 10 min. The samples above were detected using DSQ II single quadrupole GC-MS (Agilent, United States). The injection port temperature of the instrument was maintained at 250°C. The derivative sample (1 μL) was extracted and injected into the dodecyl benzene sulfonic acid column (length: 30 m × inner diameter: 250 μm × thickness: 0.25 μm). Under the direct ionization mode of 70 eV ionization energy and 8000 V accelerating voltage, the source temperature of the mass spectrometer was also maintained at 250°C. In the experiments of full scan and selective ion recording, the temperature of the quadrupole was maintained at 150°C. The initial temperature of the gas chromatograph box was set at 85°C for 5 min and then increased to 330°C at a rate of 15°C/min. Helium was used as the carrier gas with a constant flow rate of 1 mL/min, and the operating range of mass spectrometry was 50–600 m/z (Li H. et al., 2016).

### Transcriptomic Analysis of GXDK6 Under Copper Stress

Samples were cultured for 10 h and collected at −4°C. The supernatant was removed, quickly frozen with liquid nitrogen for 5 min, and quickly stored in a refrigerator maintained at −80°C. Samples were sent to the Gene *Denovo* Biotechnology Co., Ltd. (Guangzhou, China) for sequencing using the Illumina Nova Seq 6000 system.

### Malondialdehyde and Antioxidant Enzyme Activity Determination

Cells were induced to produce reactive oxygen species (ROS) under copper stress, and the oxidative damage produced by ROS could lead to cell death. MDA, which is produced due to cell aging or cell oxidative damage and peroxidation of cell membrane lipids, is an important indicator of cell oxidative damage. In this study, the thiobarbituric acid method was used to determine MDA content (Avery, 2011). MDA content, SOD activity, POD activity, CAT activity, and total antioxidant capacity were determined in accordance with the instructions of the corresponding kits for the microplate method.

### Real-Time Fluorescent Quantitative Polymerase Chain Reaction Verification of Key Differential Genes

Real-time fluorescent quantitative PCR (RT-qPCR) was used to verify the expression of key differential genes (i.e., *SOD2*, *GST*, *GSR*, and *CAT*) and the results of the transcriptome. The *ACT1* gene was used as reference. A two-step method was use for the RT-qPCR of key genes. The cDNA was synthesized from 1 mg of total RNA by using the CDNA first-strand rapid synthesis kit with the gDNA Erase in 20 mL reaction mixture. The sequences of primers for the RT-qPCR amplification of key copper-tolerant genes are listed in Table 1. The expression levels of selected genes were evaluated using the 2^–ΔΔ*Ct*^ method.

**TABLE 1:** Sequences of primers for RT-qPCR amplification of key copper-tolerant genes.

Primer name	Sequence (5′ to 3′)
*ACT*1 F	CCACCACTGCTGAGAGAGAA
*ACT1* R	GTCGGAAGGACGGAACAAAG
*GST* F	GGCACCTGCTGAATTGGAAA
*GST* R	GCAGGAACGAGTTTCTTGGA
*SOD2* F	CCGAGTCGTTGTTGAAGTCC
*SOD2* R	CAGGTCACACCATCTCCCTT
*CAT* F	GGTACCAGGTCTTGAGCCAT
*CAT* R	TTCACTGGAAGCTGCTGGTA
*GSR* F	TGGAATTGACTCCAGTGGCT
*GSR* R	CCAATCGAACCAGCTTCAGG

## Results and Discussion

### Surface Morphology of GXDK6 Under Copper Stress

**FIGURE 1:**
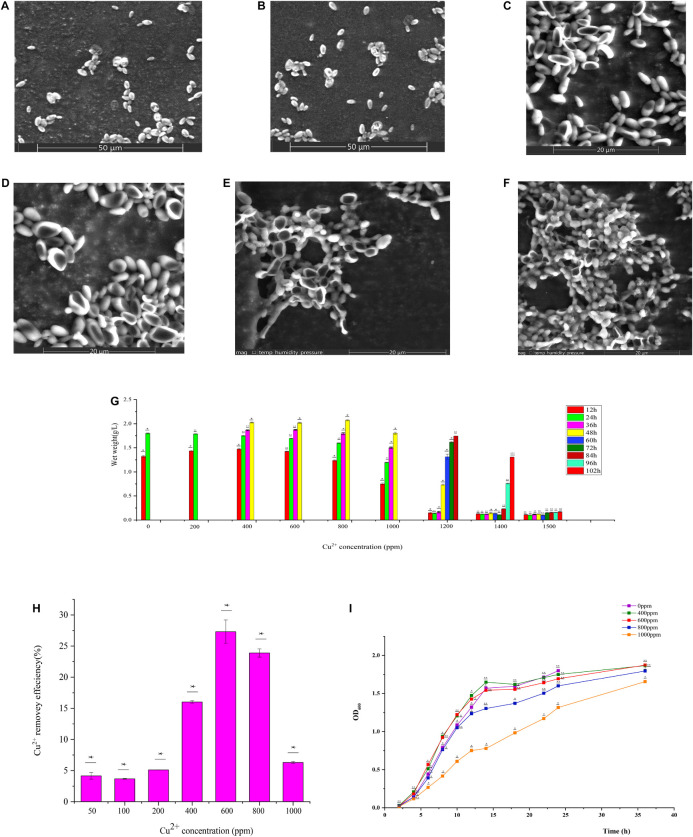
Physicochemical properties of GXDK6 incubated for **(A)** 12 h under 0 ppm copper ions, **(B)** 24 h under 0 ppm copper ions, **(C)** 12 h under 600 ppm copper ions, **(D)** 24 h under 600 ppm copper ions, **(E)** 12 h under 1000 ppm copper ions, and **(F)** 24 h under 1000 ppm copper ions. **(G)** Copper ion tolerance of GXDK6. **(H)** Copper ion removal ability of GXDK6. **(I)** Growth curve of GXDK6 under inconsistent copper ion concentrations. All experiments were carried out three times, and standard deviation analysis was carried out on the data. * Means 0.01 < *SD* < 0.05, ** means 0.001 < *SD* < 0.01, and *** means *SD* < 0.001.

GXDK6 was cultured for 12 h under the conditions of 0, 600, and 1000 ppm copper ions. The cell morphology is shown in Figure 1. In the Cu-600 group, GXDK6 cells dented in the middle and became long. In the Cu-1000 group, the degree of cell depression was high, and the size of cells decreased; in addition, some substances were attached to the cell surface (Figure 1F). These substances may be extracellular metabolites secreted by the cell, such as D-mannose, which protects the cell and improves its copper ion tolerance. These results indicate that copper stress could negatively affect the morphology of GXDK6 cells and cause cell damage.

### Copper Tolerance Range and Copper Removal Ability of GXDK6

The biomass of GXDK6 cultured at different copper ions concentrations for 102 h were measured (Figure 1G). The growth of GXDK6 was continuous under a concentration of 0–1400 ppm copper ions but was drastically inhibited when exposed to 1500 ppm copper ions. Therefore, the copper tolerance range of GXDK6 was 0–1400 ppm. The copper ions removal ability of GXDK6 was also investigated. As shown in Figure 1H, after GXDK6 was cultured with 50, 100, 200, 400, 600, 800, and 1000 ppm copper ions for 96 h, the removal efficiencies were 4.16, 3.68, 5.12, 16.01, 27.32, 23.89, and 6.31%, respectively. The removal rate increased and then decreased gradually with increasing copper ion concentration. This finding might be because copper ions could inhibit the growth of GXDK6 and decrease the biomass and removal efficiency at concentrations up to 1000 ppm. These results suggest that GXDK6 has potential application in the treatment of high-copper wastewater and the removal of copper ions in food fermentation. As shown in Figure 1I, a high-concentration of copper ions could prolong the lag phase, delay the stable phase, and decrease the thallus biomass of GXDK6.

### Whole-Genome Sequencing of GXDK6

Combined with the whole-genome sequence analysis, 11 genes (Table 2) were closely related to GXDK6 copper tolerance. Antioxidant enzyme genes *SOD2*, *PXMP4*, and *CAT* reduced cell damage by regulating the synthesis of antioxidant enzymes and the ROS induced by copper ions in the cell. Non-enzymatic antioxidant genes *GST, GSR*, *ggt*, and *gshB* encoded glutathione sulfur transferase (GST), glutathione-disulfide reductase (GSR), glutamyl transferase (GGT), and glutathione synthase (GSHB), respectively. GSR maintains high levels of GSH in cells (Deponte, 2013). The GSH/GST antioxidant system is related to the detoxification of xenobiotics, carcinogens, free radicals, and peroxides (Wahab et al., 2001; Wark et al., 2004). GST is a multi-functional detoxification enzyme that catalyzes the combination of GSH and copper to form a less toxic Cu(GS)_2_ complex and reduces the damage of copper ions to cells. In addition, *CCC2*, *CTR3*, and *FRE2* related to copper transport and reduction are the key genes for GXDK6 response to copper stress. *CCC2* encodes P-type ATPase transporter Ccc2 located in the Golgi apparatus (Yamaguchi et al., 1996) that can transport copper to the inner membrane system and under conditions of elevated extracellular copper for the efflux of copper from cells. Copper transporter3 (Ctr3), as a trimer of high-affinity copper transporter of the plasma membrane, includes three Ctr protein families in yeast (Dancis et al., 1994; Knight et al., 1996). It involves the absorption and transport of copper, which is essential for GXDK6 copper resistance.

**TABLE 2:** Annotation results of genes relevant to Cu^2+^ tolerance in *M. guilliermondii* GXDK6.

Locus	Gene symbol	Gene description	log2Cu600/Cu0	log2Cu1000/Cu0
scaffold1.g143	*SOD2*	Superoxide dismutase Fe–Mn family	–0.038883	0.498
scaffold3.g341	*PXMP4*	Peroxisomal membrane protein 4	0.52486°	0.81484
scaffold2.g396	*CAT*	Catalase	0.49006	0.010229
scaffold1.g532	*GST*	Glutathione S-transferase	0.115	0.40867
scaffold4.g64	*GSR*	Glutathione reductase	0.71881	0.887
scaffold1.g892	*gshb*	Glutathione synthase	–0.23349	0.57351
scaffold5.g379	*ggt*	Glutamyl transferase	0.8991	0.48682
scaffold1.g426	*HSP82*	ATP-dependent molecular chaperone HSP82-like	0.90718	-0.00054
scaffold9.g14	*CTR3*	Copper uptake transmembrane transporter activity	–5.9045	5.2639
scaffold3.g441	*CCC2*	Nucleoside-triphosphatase activity	1.1952	1.8073
scaffold3.g538	*FRE2*	Oxidoreductase activity	0.018021	1.1658

### Transcriptomic Analysis of GXDK6 Under Copper Stress

**FIGURE 2:**
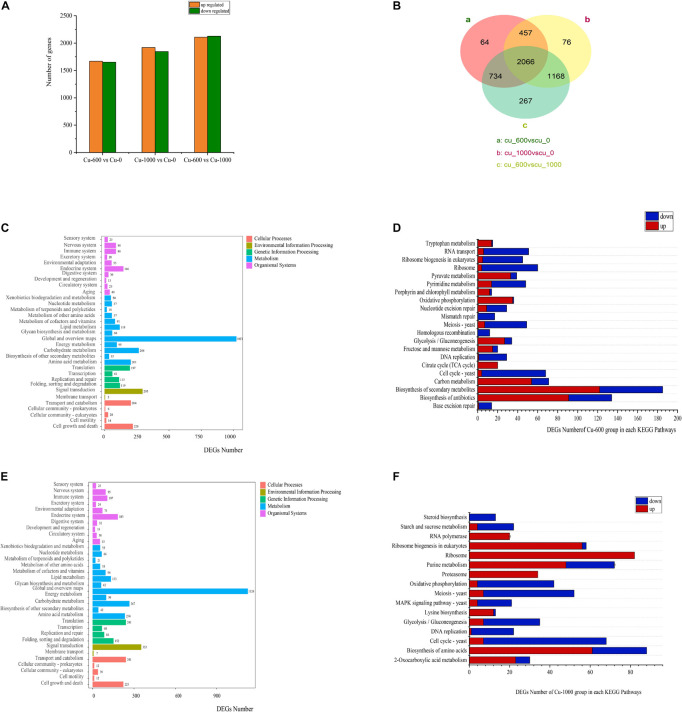
Effects of copper ions stress on GXDK6 gene transcription. **(A)** Differential genes of GXDK6 under copper ions stress for 12 h. **(B)** Venn diagram analysis of the differential genes for 12 h. **(C)** Enrichment pathway of the differential genes in the Cu-600 group for 12 h. **(D)** Significantly enriched pathway in the Cu-600 group for 12 h. **(E)** Enrichment pathway of differential genes in the Cu-1000 group for 12 h. **(F)** Significantly enriched pathway in the Cu-1000 group for 12 h.

In all groups, more than 95% of read lengths were located in *M. guilliermondii* GXDK6 (Table 3). The Q20 and Q30 in the Cu-0, Cu-600, and Cu-1000 groups were 98.03, 97.97, 97.99, 94.02, 93.88, and 93.88%, respectively. Transcriptome data results showed 3321 differentially expressed genes (DEGs) between the Cu-0 and Cu-600 groups. Compared with the Cu-0 group, the Cu-600 group had 1669 significantly upregulated genes and 1652 significantly downregulated genes. A total of 3767 DEGs (1920 significantly upregulated and 1847 significantly downregulated) were observed between the Cu-0 and Cu-1000 groups (Figure 2A). Compared with the Cu-600 group, the Cu-1000 group had 4235 DEGs (2127 upregulated and 2108 downregulated) (Figure 2B). With increasing copper ion concentration, DEGs increased, which might be because copper stress could change the expression level of some key genes of GXDK6 and certain metabolic pathways. Moreover, metabolic pathway enrichment analysis and up or down expression of DEGs (Figures 2C–F) in the pecific pathway was conducted, the DEGs of GXDK6 were related to the metabolism of fructose and mannose and the polymerization of extracellular polysaccharides. In the sulfur metabolism pathway, the key genes from hydrogen sulfide to cysteine and methionine metabolism (*MET17*, the *O*-acetylhomoserine coding gene, and *metB*, the cystathionine gamma-synthase coding gene) were significantly upregulated. This result suggested that the synthesis of cysteine and methionine upregulated, promoted the combination with copper ions, and improved the tolerance of cells to copper ions. In addition, the DEGs were related to the metabolism of porphyrin and chlorophyll, ribosome, proteasome, and ribosome biogenesis in eukaryotes.

**TABLE 3:** Statistical analysis of transcriptome sequencing genes.

Samples	Total reads	Total mapped %	Q20 %	Q30 %	GC content %
Cu-0	29282863	96.72	98.03	94.02	45.56
Cu-600	28730626	96.74	97.97	93.88	45.69
Cu-1000	27773264	95.97	97.99	93.88	45.70

### GO Annotations of Differentially Expressed Genes

**FIGURE 3:**
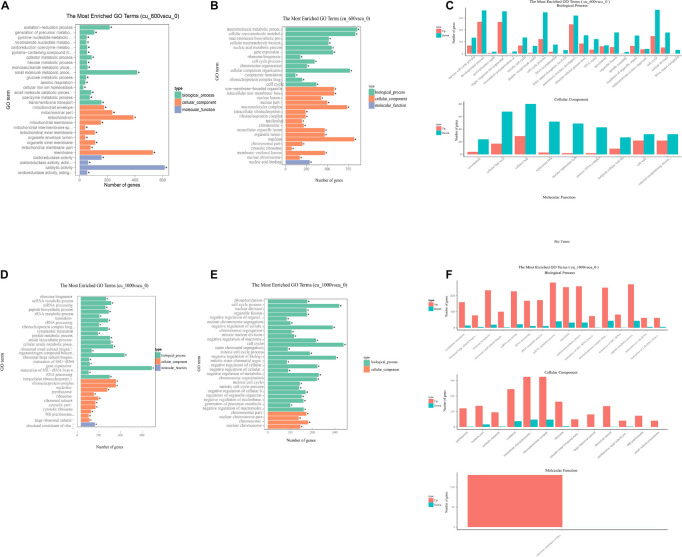
GO annotation of GXDK6 differentially expressed genes. **(A)** Upregulated GO terms in the Cu-600 group. **(B)** Downregulated GO terms in the Cu-600 group. **(C)** Upregulated and downregulated GO terms in the Cu-600 group. **(D)** Upregulated GO terms in the Cu-1000 group. **(E)** Downregulated GO terms in the Cu-1000 group. **(F)** Upregulated and downregulated GO terms in the Cu-1000 group. * Means 0.01 < p value < 0.05.

Differentially expressed genes were mapped with gene ontology (GO) terms to reveal the function of DEGs in the Cu-600 and Cu-1000 groups. All DEGs were annotated into three major categories: biological process, cellular component, and molecular function. Regardless of group, DEGs were remarkably enriched in biological process and cellular component. In this study, as shown in Figure 3, the 30 most enriched GO items were selected for display. The remarkably enriched GO terms were meiotic cell cycle process (204 DEGs), developmental process (632 DEGs), and postreplication repair (23 DEGs) in the Cu-600 group and ribonucleoprotein complex (293 DEGs), ncRNA processing (249 DEGs), peptide biosynthetic process (290 DEGs), and non-membrane-bound organelles (842 DEGs) in the Cu-1000 group. Among the GO items, oxidation–reduction process (217DEGs) and oxidoreductase activity (159 DEGs) were remarkably upregulated in the Cu-600 group. This result indicates that under copper stress, the redox process and oxidoreductase activity play important roles in GXDK6 copper tolerance. The oxidation or reduction of heavy metals is the key process for heavy metal detoxification (Dang et al., 2021). In addition, non-membrane-bound organelles (581 DEGs) and intracellular non-membrane-bound organelles (581 DEGs) were remarkably downregulated items in the Cu-600 group. Non-membrane-bound organelles are described as a dynamic structure that usually exhibits liquid-like physical properties (Brangwynne et al., 2009, 2011). Although non-membrane-bound organelles are involved in important biological processes, their precise role remains elusive and is usually associated with multiple functional pathways (Mitrea and Kriwacki, 2016). The protein composition and morphology of non-membrane organelles change in response to the cellular environment. This ability to respond to environmental cues reflects the mechanism of non-membrane organelles to participate in stress sensing (Boisvert et al., 2007; Lallemand-Breitenbach and de Thé, 2010; Buchan, 2014). In the Cu-1000 group, among all the upregulated GO items, ribosome biogenesis (233 DEGs), intracellular ribonucleoprotein complex (323 DEGs), and ribonucleoprotein complex (323 DEGs) were remarkably upregulated. This result indicates that copper stress can stimulate protein synthesis. Under copper ion stress, cells relieve stress by promoting the synthesis of proteins with specific functions, such as heat shock proteins (HSPs), and repairing damaged proteins or removing them to restore protein homeostasis (Hartl, 1996). Copper ion stress inhibited cell meiosis, which may be beneficial to copper stress response by decreasing the frequency of genetic information errors. The GO annotation results showed that these DEGs are involved in the regulation of gene transcription, translation, post-translational modification, and signal molecular transmission during cell development and respond to copper stress by regulating the synthesis and transport of metabolites. Annotation results indicated that in GXDK6, copper stress affected the expression of related key DEGs, and DEGs affected the split, growth, development, and death of cells in response to copper stress. We speculated that GXDK6 increases the synthesis of various proteins and promotes respiration to resist copper stress under high-copper conditions. In addition, the increase in respiration rate may be due to the increase in energy levels and the redox balance to the reduced cell state. It also provides fuel for the transcription and translation of protein and enzyme activities. The copper tolerance of GXDK6 was revealed at the transcriptomic level, but the underlying mechanism remains unclear. The molecular mechanism of GXDK6’s tolerance to copper stress at the metabolomic level should be further elucidated.

### Metabolic Pathway Analysis of GXDK6

The KEGG enrichment analysis of DEGs was performed to clarify the regulation network further. In the Cu-600 and Cu-1000 groups, DEGs related to proteasome and peroxisome were annotated. The expression levels of most genes involved in the proteasome and peroxisome were upregulated in the Cu-600 and Cu-1000 groups (Tables 4, 5). A peroxisome is a type of organelle involved in fatty acid oxidation, amino acid and polyamine catabolism, and ROS reduction (Bonekamp et al., 2009). Peroxisome is a highly conserved cellular pathway that plays an important role in the selective degradation of cellular proteins, thereby helping regulate various vital cellular functions (Banfalvi, 2011). The enhanced proteasome activity is beneficial to reduce copper stress-induced protein ubiquitination and carbonylation (Romero-Puertas et al., 2004), which is advantageous for GXDK6 to survive under high-copper concentrations. Therefore, peroxisome and proteasome play important roles in reducing the carbonylation and ubiquitination of proteins induced by copper and maintain the stability of intracellular proteins, thus improving the tolerance of GXDK6 to copper. In the Cu-1000 group, ribosome (82 DEGs, Supplementary Table S1) and ribosome biogenesis in eukaryotes (56 DEGs) were remarkably upregulated. This finding was consistent with the GO annotation. GXDK6 could promote the upregulation of ribosomes under copper stress, thereby promoting the expression of various functional proteins and helping GXDK6 against high-copper stress. The remarkably enriched pathways, including fructose and mannose metabolism, carbon metabolism, and biosynthesis of secondary metabolites, were selected to analyze the regulation mechanism. These metabolic pathways were remarkably enriched and upregulated in the Cu-600 and Cu-1000 groups. In the Cu-600 group, 20 DEGs were enriched in the metabolism of fructose and mannose (Supplementary Table S2). The metabolic pathway analysis of fructose and D-mannose (Supplementary File S2) showed that these genes are involved in the regulation of GXDK6 fructose and mannose metabolism. Among these genes, the most upregulated gene was *SORD* (scaffold2.g957, upregulated by 2.1307 fold), which encodes L-iditol 2-dehydrogenase, followed by *LRA1*, which encodes L-rhamnose 1-dehydrogenase (scaffold4.g291, upregulated by 1.837 fold). The most downregulated gene in this pathway was *FBA* (scaffold4.g508, downregulated by 1.34 fold). These results indicate that *LRA1*, *SORD*, and *FBA* are the key genes in the metabolic pathways of fructose and mannose. The significant enrichment of this metabolic pathway helped GXDK6 survive under high-copper concentrations, indicating that fructose and mannose are beneficial for GXDK6 to improve its copper resistance. In the Cu-1000 group, four DEGs were enriched in the metabolic pathway of fructose and mannose. The most upregulated gene was *LRA1*, followed by *SORD*, which were upregulated by 2.29 and 2.23 folds, respectively. By contrast, the most downregulated gene was *HK* (scaffold5.g418, downregulated by 2.21 fold). Therefore, *LRA1*, *SORD*, *FBA*, and *HK* are the key genes that regulate the metabolism of fructose and mannose. Hameed et al. (2014) demonstrated that D-mannose can enhance the activities of SOD, POD, and ascorbate peroxidase antioxidants in cells and the process of cell apoptosis by affecting the antioxidant defense system of cells. In addition, polysaccharides such as fructose and mannose are secreted outside the cell to form extracellular polymers (Panosyan et al., 2018), which chelate with metal ions to prevent the access of metal ions to the cell, thereby protecting the cell (Hou et al., 2017). In the Cu-1000 group, the product with the highest relative content after 24 and 48 h of fermentation was D-mannose, and the extracellular polymer on the cell surface is shown in Figure 1F. These findings suggest that the metabolism of fructose and mannose could support GXDK6 survival under high-copper conditions and that the accumulated metabolites of D-mannose contribute to the copper tolerance of GXDK6.

**TABLE 4:** Differentially expressed genes involved in proteasome metabolism (KEGG: pgu03050) in the Cu-600 and Cu-1000 groups.

Locus	Gene symbol	Gene description	log2Cu600/Cu0	log2Cu1000/C0
scaffold1.g443	*PSMB2*	20S proteasome subunit beta 4	0.091223	1.2583
scaffold1.g451	*PSMD12*	26S proteasome regulatory subunit N5	0.45014	0.87536
scaffold1.g666	*PSMD3*	26S proteasome regulatory subunit N3	0.12085	0.55595
scaffold1.g685	*PSMD6*	26S proteasome regulatory subunit N7	0.02119	0.72661
scaffold1.g857	*PSMA7*	Proteasome subunit alpha 4	0.33304	1.0924
scaffold1.g871	*PSMB4*	20S proteasome subunit beta 7	0.37555	0.37395
scaffold1.g940	*PSMD14*	RPN11, POH1; 26S proteasome regulatory subunit N11	0.59417	1.4697
scaffold2.g156	*PSMD8*	26S proteasome regulatory subunit N12	0.034602	1.0235
scaffold2.g422	*PSMA4*	Proteasome subunit alpha 3	0.07629	1.071
scaffold2.g507	*PSMD1*	26S proteasome regulatory subunit N2	0.19362	1.0202
scaffold2.g609	*PSMA2*	20S proteasome subunit alpha 2	0.26388	0.94098
scaffold2.g860	*PSMD4*	26S proteasome regulatory subunit N10	0.012911	1.1105
scaffold3.g173	*PSMC4*	26S proteasome regulatory subunit T3	0.24308	1.1136
scaffold3.g241	*PSMB7*	20S proteasome subunit beta 2	0.63464	0.41087
scaffold3.g511	*PSMD7*	26S proteasome regulatory subunit N8	–0.019271	1.117
scaffold3.g752	*PSMD9*	26S proteasome regulatory subunit N4	0.55708	0.52574
scaffold3.g800	*PSMD2*	26S proteasome regulatory subunit N1	–0.21873	0.76283
scaffold4.g227	*PSMA6*	20S proteasome subunit alpha 1	0.25651	1.2642
scaffold4.g296	*PSMB6*	20S proteasome subunit beta 1	0.29356	1.1496
scaffold4.g431	*PSMC5*	26S proteasome regulatory subunit T6	0.62085	1.0636
scaffold4.g500	*PSMC6*	26S proteasome regulatory subunit T4	0.49906	1.2245
scaffold5.g134	*PSMB3*	20S proteasome subunit beta 3	0.34692	0.81118
scaffold5.g179	*PSMD13*	26S proteasome regulatory subunit N9	0.021832	1.0789
scaffold5.g294	*RPN13*	26S proteasome regulatory subunit N13	0.5749	0.70311
scaffold5.g66	*PSMC1*	26S proteasome regulatory subunit T2	0.27117	1.1258
scaffold6.g136	*PSMC3*	26S proteasome regulatory subunit T5	0.13119	1.6548
scaffold6.g137	*SHFM1*	26S proteasome complex subunit DSS1	0.4518	0.27674
scaffold6.g375	*PSMA5*	20S proteasome subunit alpha 5	0.5201	1.0814
scaffold6.g460	*PSMB5*	20S proteasome subunit beta 5	0.034939	1.0986
scaffold7.g119	*PSMC2*	26S proteasome regulatory subunit T1	0.60002	1.2257
scaffold7.g167	*POMP*	Proteasome maturation protein	0.3478	0.44275
scaffold7.g355	*PSME4*	Proteasome activator subunit 4	–0.078303	0.33056
scaffold8.g59	*PSMA3*	20S proteasome subunit alpha 7	0.21905	0.9215
scaffold9.g19	*PSMB1*	20S proteasome subunit beta 6	0.43369	0.76543
scaffold9.g63	*PSMD11*	26S proteasome regulatory subunit N6	0.26589	1.3487
scaffold9.g80	*PSMA1*	20S proteasome subunit alpha 6	0.39463	1.1471

**TABLE 5:** Differentially expressed genes involved in peroxisome metabolism (KEGG: pgu01230) in the Cu-600 and Cu-1000 groups.

Locus	Gene symbol	Gene description	log2Cu600/Cu0	log2Cu1000/C0
scaffold1.g1010	*PXA*	ATP-binding cassette, subfamily D (ALD), peroxisomal long-chain fatty acid import protein	0.4229	–0.14307
scaffold1.g1018	*DECR2*	2,4-dienoyl-CoA reductase [(3E)-enoyl-CoA-producing], peroxisomal	0.58534	0.15645
scaffold1.g1053	*ACOX1*	Acyl-CoA oxidase	0.24958	–1.9131
scaffold1.g143	*SOD2*	Superoxide dismutase, Fe–Mn family	–4.3844	–5.3848
scaffold1.g304	*PEX1*	Peroxin-1	–0.23685	–0.52134
scaffold1.g54	*PEX14*	Peroxin-14	0.8349	0.25489
scaffold1.g563	*SOD2*	Superoxide dismutase, Fe–Mn family	–0.038883	0.4982
scaffold1.g566	*ACOX1*	Acyl-CoA oxidase	0.87157	0.25607
scaffold1.g567	*ACOX1*	Acyl-CoA oxidase	0.62598	−1.2455
scaffold1.g59	*DECR2*	2,4-dienoyl-CoA reductase [(3E)-enoyl-CoA-producing], peroxisomal	1.0262	–1.0562
scaffold1.g632	*PEX6*	Peroxin-6	–0.18241	–1.3067
scaffold1.g676	*DAO*	D-amino-acid oxidase	1.1432	0.22924
scaffold1.g76	*ECI2*	Delta3-Delta2-enoyl-CoA isomerase	0.85093	-0.51584
scaffold2.g123	*PXA*	ATP-binding cassette, subfamily D (ALD), peroxisomal long-chain fatty acid import protein	0.70812	0.97567
scaffold2.g314	*PEX19*	Peroxin-19	0.21879	−0.14506
scaffold2.g396	*CAT*	Catalase	0.49006	0.010229
scaffold2.g483	*ACSL*	Long-chain acyl-CoA synthetase	–0.39113	0.027016
scaffold2.g541	*ACSL*	Long-chain acyl-CoA synthetase	0.23803	0.95725
scaffold2.g697	*PEX5*	Peroxin-5	1.0284	1.1581
scaffold2.g76	*PEX7*	Peroxin-7	0.5191	–0.33172
scaffold2.g879	*ECH1*	Delta3,5-Delta2,4-dienoyl-CoA isomerase	0.4061	–0.13587
scaffold2.g886	*ACAA1*	Acetyl-CoA acyltransferase 1	0.046079	–0.79773
scaffold2.g925	*ACAA1*	Acetyl-CoA acyltransferase 1	1.4234	0.94433
scaffold3.g106	*SOD2*	Superoxide dismutase, Fe–Mn family	–0.18127	0.26071
scaffold3.g200	*SOD2*	Superoxide dismutase, Fe–Mn family	0.84481	–0.96481
scaffold3.g245	*ACSL*	Long-chain acyl-CoA synthetase	0.096248	–0.15418
scaffold3.g330	*IDH1*	Isocitrate dehydrogenase	0.96882	0.69492
scaffold3.g341	*PXMP4*	Peroxisomal membrane protein 4	0.52486	0.81484
scaffold3.g447	*AGXT*	Alanine-glyoxylate transaminase/serine-glyoxylate transaminase/serine-pyruvate transaminase	–0.26587	0.88446
scaffold3.g66	*PEX3*	Peroxin-3	–0.041963	–0.0034226
scaffold4.g351	*IDH1*	Isocitrate dehydrogenase	0.059291	–0.28133
scaffold5.g380	*CRAT*	Carnitine *O*-acetyltransferase	0.76987	3.0562
scaffold5.g412	*ACSL*	Long-chain acyl-CoA synthetase	0.30298	–0.70738
scaffold5.g69	*PEX2*	Peroxin-2	0.3698	0.33352
scaffold6.g113	*PMP34*	Solute carrier family 25 (peroxisomal adenine nucleotide transporter), member 17	–0.17095	–0.29614
scaffold6.g413	*MVK*	Mevalonate kinase	–0.17485	0.52333
scaffold6.g427	*AGXT*	Alanine-glyoxylate transaminase/serine-glyoxylate transaminase/serine-pyruvate transaminase	0.23625	−0.68054
scaffold6.g452	*MPV17*	Protein Mpv17	-0.25531	1.1494
scaffold7.g227	*CRAT*	Carnitine *O*-acetyltransferase	0.16242	–0.41137
scaffold7.g387	*CRAT*	Carnitine *O*-acetyltransferase	0.17573	–0.4754
scaffold7.g50	*PEX13*	Peroxin-13	0.25115	0.32138
scaffold7.g73	*CAT*	Catalase	0.41153	–3.8607
scaffold8.g82	*NUDT12*	NAD + diphosphatase	0.95423	0.72384
scaffold9.g93	*HAO*	(S)-2-hydroxy-acid oxidase	1.6205	1.0247

For carbon metabolism, 71 DEGs were remarkably enriched in the Cu-600 group. These genes were involved in the regulation of the glycolytic pathway, pentose phosphate pathway, citrate cycle, and some methane metabolic pathways. In the Cu-600 group, the most upregulated gene was *HAO* (scaffold9.g93, upregulated by 1.62 fold), which encodes (S)-2-hydroxy-acid oxidase and participates in GXDK6 glyoxylate regulation, dicarboxylate metabolism, secondary metabolite biosynthesis, carbon metabolism, and peroxisome metabolism. By contrast, the most downregulated gene was *GAPDH* (scaffold9.g10, downregulated by 2.787 fold), which encodes glyceraldehyde-3-phosphate dehydrogenase and participates in glycolysis regulation, secondary metabolite biosynthesis, carbon metabolism, and amino acid biosynthesis. *GAPDH* expression is inhibited, which is the protective mechanism of GXDK6 under heavy metal stress. Krobitsch and colleagues (Ralser et al., 2007) provided the first direct evidence that the oxidation inhibition of glycolytic enzymes (including GAPDH) is a controlled reaction that enables cells to redirect their carbohydrate flux from glycolysis to the pentose phosphate pathway, generating NADPH, the reducing agent that protects cells from oxidative stress. In the Cu-1000 group, 38 differential genes in the carbon metabolic pathway were remarkably enriched, and the most upregulated gene was the formamidase-encoding gene *AFMID* (scaffold5.g9, upregulated by 2.196 folds), which is involved in the regulation of cyanoamino acid metabolism, glyoxylate and dicarboxylate metabolism, nitrogen metabolism, and carbon metabolism. The regulation of those enzymes can be related to the recycling of amino acids for further protein synthesis. The most downregulated gene was still *GAPDH* (scaffold9.g10, downregulated by 5.456 folds). These results indicate that when the copper ion concentration reaches 1000 ppm, *HAO*, *GAPDH*, and *fmdA* are the key genes in the GXDK6 carbon metabolic pathway and that the carbon metabolism regulated by *HAO*, *GAPDH*, or *fmdA* supports GXDK6 to survive under high-copper stress.

In the Cu-600 group, 185 DEGs were remarkably enriched in the biosynthesis of secondary metabolites. The most upregulated gene was *RIB3* (scaffold3.g378, upregulated by 1.97 folds), and the most downregulated gene was *GAPDH* (scaffold9.g10, downregulated by 2.79 folds). These DEGs are involved in the regulation of carbon metabolism riboflavin metabolism, biosynthesis of secondary metabolites, biosynthesis of cofactors, glycolysis/gluconeogenesis, and biosynthesis of amino acid metabolism. In the Cu-1000 group, 111 genes were enriched in the biosynthesis of secondary metabolites. The most upregulated gene was *ARG* (scaffold3.g109, upregulated by 3.99 folds), which participates in arginine biosynthesis regulation, arginine and proline metabolism, secondary metabolite biosynthesis, and amino acids. The most downregulated gene was *GAPDH* (scaffold9.g10, downregulated by 5.46 folds). Results showed that *RIB3*, *GAPDH*, and *ARG* are the key genes of secondary metabolite synthesis. In plants, the stress of heavy metals induces the biosynthetic pathway by stimulating the immune response in glutaraldehyde and causes the accumulation of secondary metabolites (Lajayer et al., 2017). Therefore, copper stress would stimulate the synthesis of various secondary metabolites that help GXDK6 survive under high-copper conditions. In addition, the biosynthesis of amino acids (Supplementary Table S3), especially proline, has a positive effect on the response to heavy metals (Xu et al., 2012). The proline in the transmembrane helical segment plays a specific role in the structure and function of membrane proteins, such as some molecular chaperones for membrane stabilization (Deber and Therien, 2002). Proline accumulation is considered to be an adaptive effect in response to environmental stress, and this accumulation mainly depends on increased synthesis and decreased degradation (Verbruggen and Hermans, 2008). Proline plays an important role in protecting cell homeostasis from stress damage (Li et al., 2015). In the carbon metabolic pathway and the biosynthesis of secondary metabolites, the *GAPDH* gene was involved in metabolic regulation and was the most downregulated in the Cu-1000 and Cu-600 groups. This result indicates that *GAPDH* is the key gene in the regulatory network of GXDK6 carbon metabolism and biosynthesis of secondary metabolites. Thus, this gene contributes remarkably to the copper resistance of GXDK6.

### Antioxidant Enzyme Gene Expression Analysis

In this study, antioxidant enzyme genes, including *SOD2*, *PXMP4*, and *CAT*, were selected to analyze the contribution of antioxidant enzyme genes to GXDK6 copper tolerance. In the Cu-600 group, the expression levels of *PXMP4* and *CAT* were remarkably upregulated, and the expression of *SOD2* showed no significant change. In the Cu-1000 group, the expression levels of *PXMP4*, *SOD2*, and *CAT* were remarkably upregulated. Antioxidant enzymes acted as the main scavenger of copper-induced ROS, and SOD decomposed O^2–^ into oxygen or hydrogen peroxide. When cells were exposed to high concentrations of copper ions (Cu-600), *SOD2* expression was remarkably upregulated, enhancing ROS decomposition and reducing cell toxicity. However, hydrogen peroxide is still toxic to cells and can be broken down by *CAT* and *POD* (Li et al., 2020), thereby reducing H_2_O_2_ in cells. The increased expression of antioxidant enzyme genes can help improve the tolerance of GXDK6 to copper. The enhanced expression of HSP82 also contributes to cell protection from copper stress (Li C. et al., 2018).

### Analysis of Non-enzymatic Antioxidant Gene Expression

When copper is transported to cells, copper induces the synthesis of the soluble tripeptide (GSH) present in yeast, which can form Cu(GS)_2_ with copper under the catalysis of GST, and reduces the toxicity of heavy metals to cells (Dourado et al., 2008). GST, which is widely distributed in microbial cells and has a detoxification effect on heavy metals, can increase the resistance of microbes to heavy metals and maintain the normal metabolism of cells when exposed to endogenous or exogenous harmful compounds (Ezaki et al., 2008). KEGG pathway analysis also showed that the glutathione metabolic pathway gene expression levels in the Cu-600 and Cu-1000 groups changed remarkably (Supplementary Table S4). This pathway included 28 DEGs, and the expression levels of most genes, including *GGT*, *GST, GSR, GSHB*, and *GPX*, were upregulated under copper ion conditions. *GST*, *GSR*, and *GGT* encoded GST, GSR, and GGT, respectively. GSH and GST play key roles in copper ion detoxification. GST catalyzed the combination of GSH and copper to form a less toxic Cu(GS)_2_ complex, which could reduce the damage of copper ions to cells. In this study, *GST* and *GSR* were remarkably upregulated in the Cu-600 and Cu-1000 groups. *GGT* was upregulated in the Cu-600 group and downregulated in the Cu-1000 group. The increased expression levels of *GST*, *GSR*, and *GGT* could contribute to cell tolerance to copper and provide an effective defense against copper ion-induced oxidative stress. The expression levels of most differential genes in the metabolic pathways of fructose and mannose were upregulated. In the Cu-600 group, 15 genes in the metabolic pathways of fructose and mannose were remarkably upregulated (20 background genes).

KEGG enrichment analysis also revealed that the expression of *HSP82* was remarkably upregulated in the Cu-1000 group but did not remarkably change in the Cu-600 group. HSPs are important proteins commonly found as chaperones that mediate the correct assembly of proteins and their intracellular location to protect other proteins against chemical and physical changes in the cytoplasm (Lanneau et al., 2008). HSPs constitute approximately 5–10% of the total protein of all normal unstressed cells, perform many constitutive stabilization state functions, and play an important role in maintaining cell homeostasis (Chiricosta et al., 2020). In stress-free cells, HSPs play various instinctive functions. However, when cells face stress (e.g., heat, acid–base, heavy metal stresses), the HSP synthesis increases. In this study, in the Cu-1000 group, the expression of *HSP82* significantly increased. Increased expression of *HSP82* reduced cell damage and helped cells against various adverse environments.

In addition, RNA-Seq results showed that other genes related to copper tolerance changed significantly. Copper ion-transporting P-type ATPase (Ccc2) was required for the export of copper from the cytosol into the extracytosolic compartment. *CCC2* expression was remarkably upregulated in the Cu-1000 group and downregulated in the Cu-600 group. This finding might be because under high-concentration copper ions (Cu-1000), GXDK6 enhanced the expression of this gene to improve the efficiency of transporting intracellular copper ions into the inner membrane system, which was conducive to the effective absorption and degradation of copper ions and reduced the damage of copper ions to cells. In addition, the expression levels of *CTR3* and *FRE2* changed significantly. *CTR3* was significantly downregulated in the Cu-1000 and Cu-600 groups. By contrast, *FRE2* was significantly upregulated in the Cu-1000 and Cu-600 groups. Ctr3 and Fre2p were the proteins related to copper transport, and the upregulation or downregulation of their expression might lead to changes in the tolerance of GXDK6 to copper. Under copper stress, the expression levels of Ctr3 in the Cu-600 and Cu-1000 groups were remarkably downregulated, which reduced the uptake of copper ions by GXDK6, whereas *FRE2* was remarkably upregulated, which increased the copper ion reduction efficiency and reduced the toxicity of copper ions to cells. This result revealed that the synergistic effect of these two proteins reduced copper ions to monovalent copper ions and transported copper into the intracellular membrane system, thereby reducing the toxicity of copper ions to cells and improving the tolerance of cells to copper.

### Metabolomics Analysis of GXDK6 Under Copper Stress

**FIGURE 4:**
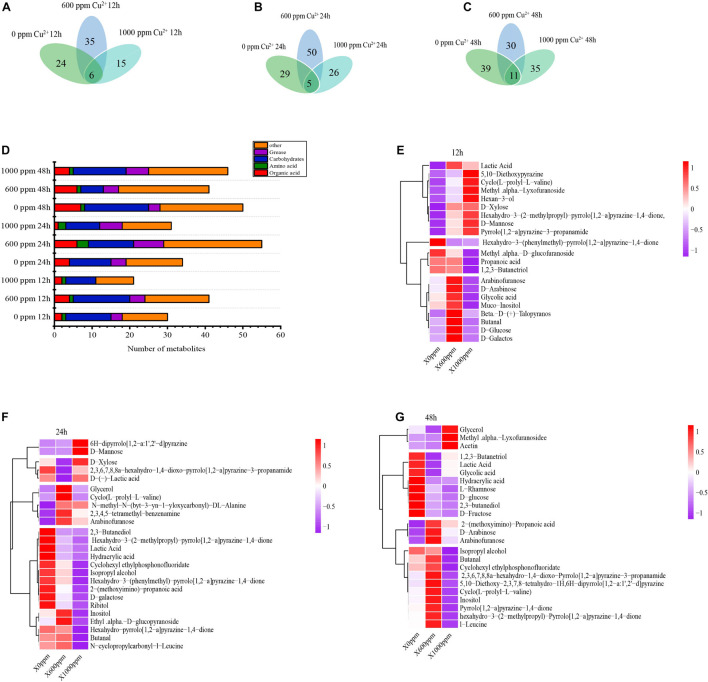
Effects of copper ion stress on the metabolism of GXDK6. Metabolites of GXDK6 under copper ions stress for **(A)** 12, **(B)** 24, and **(C)** 48 h. **(D)** Classification analysis of metabolites. Heatmap analysis of differential metabolites under copper ions tress for **(E)** 12, **(F)** 24, and **(G)** 48 h.

When GXDK6 was cultured at copper ion concentrations of 0, 600, and 1000 ppm for 24 h, the types of metabolites changed significantly (Figure 4). These metabolites could be divided into six categories, namely, sugars, alcohols, phenols, organic acids, lipids, and other organic substances. The Cu-0, Cu-600, and Cu-1000 groups were detected with 30, 41, and 21 metabolites, respectively, at a fermentation time of 12 h; 34, 55, and 31 fermentation products, respectively, at a fermentation time of 24 h; and 50, 41, and 46 fermentation products, respectively, at a fermentation time of 48 h. D-mannose and glycerol were common products, indicating that regardless of copper concentration, some certain metabolic pathways were conservative.

Metabolite heat map analysis (Figures 4E–G) showed differences in metabolite expression among the different groups, and the overview of metabolite upregulation or downregulation among the different groups could be identified. For example, at a fermentation time of 24 h, 19, 25, and 7 carbohydrates were detected in the Cu-0, Cu-600, and Cu-1000 groups, respectively. With increasing copper ions concentration, the carbohydrate production increased significantly in the Cu-600 group and weakened in the Cu-1000 group, indicating that copper ions would affect the carbohydrate metabolism of GXDK6. In addition, at a fermentation time of 24 h, the concentration of lipids increased with increasing copper ion concentration. The regulation of lipid metabolism is related to membrane regeneration (Pike, 2003). These results indicate that the accumulation of oil helps protect cells and enhance GXDK6 resistance to copper stress. At fermentation times of 24 and 48 h, the products with the highest relative content were 2,3-butanediol in the Cu-0 group, glycerin in the Cu-600 group, and D-mannose in the Cu-1000 group. This result indicates that the increased copper ion concentration stimulates GXDK6 to accumulate D-mannose, which is an important product involved in regulating GXDK6 copper tolerance. In the Cu-600 and Cu-1000 groups, the addition of 0.1–0.3% and 0.1–0.6% D-mannose exogenously increased the copper-tolerant survival rate of GXDK6 (Supplementary Figure S2). In this study, D-mannose was related to the copper stress tolerance of GXDK6 and played an important role in improving the copper resistance of GXDK6. D-mannose is likely an important substance for GXDK6 to respond to copper stress at the metabolite level.

### Cell Damage Identification and Enzyme Activity Determination

**FIGURE 5:**
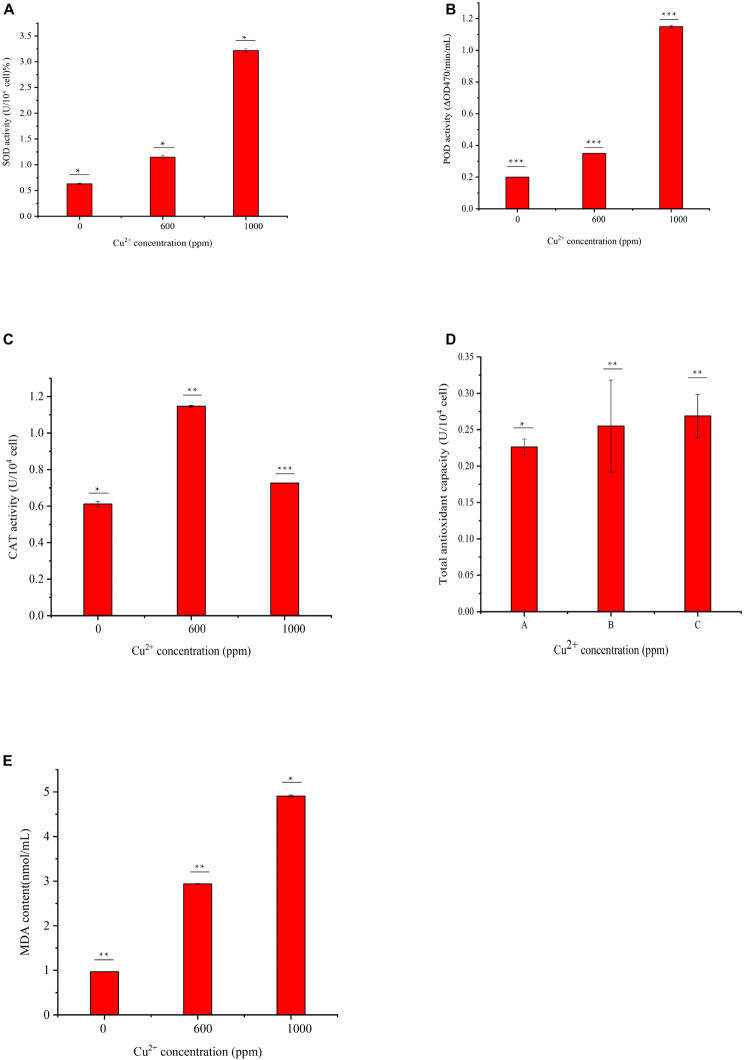
Determination of antioxidant enzyme activities and MDA content of GXDK6 in different copper ion concentrations: **(A)** SOD activity, **(B)** POD activity, **(C)** CAT activity, **(D)** total antioxidant capacity, and **(E)** cell MDA content. All experiments were carried out three times, and standard deviation analysis was carried out on the data. * Means 0.01 < *SD* < 0.05, ** means 0.001 < *SD* < 0.01, and *** means *SD* < 0.001.

**FIGURE 6:**
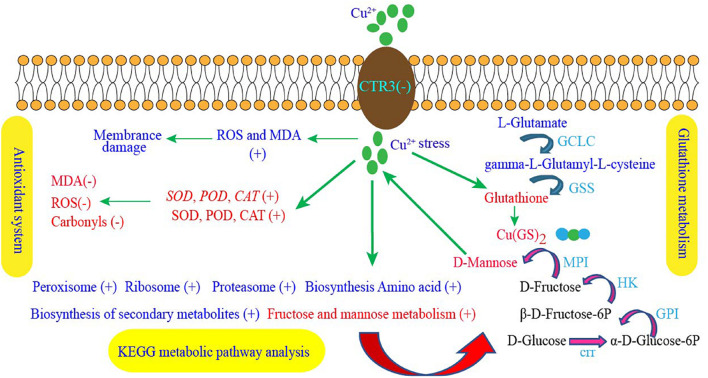
GXDK6 copper ion tolerance mechanism. “+” Indicates that the metabolic pathway, gene expression, enzyme activity, or substance content significantly upregulated under copper stress, whereas “–” indicates that the gene expression, enzyme activity, or substance content significantly downregulated compared with that in the copper ion-free group.

In this study, the MDA contents of cells exposed to 600 and 1000 ppm copper ions were measured. As shown in Figure 5E, the contents of MDA at the Cu-600 and Cu-1000 groups were 2.94 and 4.91 nmol/mL, respectively, which were evidently higher than that in Cu-0 group (0.97 nmol/mL). Results showed that copper stress induced ROS production, causing a large amount of MDA production and leading to cell damage. Enzyme activity determination results are shown in Figure 5. The SOD enzyme activity (Figure 5A) in the Cu-1000 group was the highest and 5.1 folds higher than that in the Cu-0 group. The enzyme activity of the Cu-600 group was 1.82 folds that of the Cu-0 group. Similar to the SOD activity, the POD activity (Figure 5B) in the Cu-1000 group was the highest and was 5.75 times higher than that in the Cu-0 group. The activity of POD in the Cu-600 group was 1.75 folds that of the Cu-0 group; in the Cu-600 and Cu-1000 groups, the CAT activity (Figure 5C) and total antioxidant capacity (Figure 5D) were higher than that in the Cu-0 group. These results showed that the activities of antioxidant enzymes were significantly higher under copper ion stress than under no copper ion stress. This result suggests that the elevated antioxidant enzyme activities induced by copper ion stress contribute to decreasing copper ion-induced ROS generation, oxidative damage, and cell death, leading to improved copper tolerance. As Figure 6 showed, the copper tolerance the antioxidant enzyme system, glutathione metabolism and metabolic pathways such as fructose and mannose metabolism play a key role in GXDK6 copper tolerance.

### Verification of Gene Expression

The SYBR Green RT-qPCR was used to validate the expression of genes related to copper tolerance selected by RNA-Seq. As shown in Supplementary Figure S3, the expression levels of *CAT*, *GST*, and *GLR1* in the Cu-1000 group were upregulated by 2.67, 1.41, and 2.96 folds, respectively, and no significant difference was observed in *SOD2* expression. These results are consistent with the transcriptome data, indicating high reliability of the RNA-seq analysis.

## Conclusion

The copper tolerance mechanism of *M. guilliermondii* GXDK6 plays an important role in removing copper ions in the microbial food fermentation industry. This study used integrated omics technology to reveal the copper tolerance mechanism of *M. guilliermondii* for the first time. The absorption and transport of copper ions and the combination of glutathione with copper ions played an important role in the detoxification of copper ions. Antioxidant enzymes SOD, CAT, and POD eliminated copper ion-induced ROS and reduced cell damage. *AFMID*, *HK*, and *GAPDH* were considered as new genes of *M. guilliermondii* GXDK6 related to copper tolerance. D-mannose contributed to GXDK6 copper tolerance. This study provides new insights for studying the molecular regulation mechanism of copper tolerance in *M. guilliermondii* and contributes to the removal of copper ions in the food fermentation industry.

## Data Availability Statement

This Whole Genome Shotgun project has been deposited at DDBJ/ENA/GenBank under the accession JAIGNZ000000000. The version described in this manuscript is version JAIGNZ010000000. This Whole Genome Shotgun project has been deposited at DDBJ/ENA/GenBank under the accession JAIGNZ000000000. The version described in this manuscript is version JAIGNZ010000000. The transcriptome data under the copper ion stress of GXDK6 has been uploaded to the SRA database of the NCBI database, the accession number is PRJNA752222, visit the website https://www.ncbi.nlm.nih.gov/Traces/study/?acc=PRJNA752222 to obtain the data details.

## Author Contributions

RB conducted the experiments and wrote the manuscript. BY conducted the experiments and provided the theoretical direction of multi-omics research. HS conducted the experiments and revised the manuscript. MZ and HB arranged and analyzed the experimental data. XC and XM provided the technical support. GS corrected the language errors of the manuscript. CJ designed the study. All authors read and approved the final manuscript.

## Conflict of Interest

CJ was employed by company Guangxi Flyment Biotechnology Co. Ltd. The remaining authors declare that the research was conducted in the absence of any commercial or financial relationships that could be construed as a potential conflict of interest.

## Publisher’s Note

All claims expressed in this article are solely those of the authors and do not necessarily represent those of their affiliated organizations, or those of the publisher, the editors and the reviewers. Any product that may be evaluated in this article, or claim that may be made by its manufacturer, is not guaranteed or endorsed by the publisher.
