# Supplementary material for: Copper Tolerance Mechanism of the Novel Marine Multi-Stress Tolerant Yeast Meyerozyma guilliermondii GXDK6 as Revealed by Integrated Omics Analysis
Source: Front Microbiol. 2021 Nov 18;12:771878. doi: 10.3389/fmicb.2021.771878 (PMC8637192; doi:10.3389/fmicb.2021.771878)

Additional file 1. Transcriptome analysis of GXDK6 under copper ions stress

Table S1. DEGs involved in the Ribosome metabolism (KEGG: pgu03010) in Cu-600 and Cu-1000 groups.

Locus	Gene symbol	Gene description	log2Cu600/Cu0	log2Cu1000/C0
Novel00002	*RP-S29e*	small subunit ribosomal protein S29e	-0.25481	0.8833
scaffold1.g1024	*RP-L26e*	large subunit ribosomal protein L26e	-1.0631	0.63323
scaffold1.g1034	*RP-L10e*	large subunit ribosomal protein L10e	0.48597	1.6414
scaffold1.g233	*RP-L5*	large subunit ribosomal protein L5	-0.27649	0.68581
scaffold1.g323	*RP-S16*	small subunit ribosomal protein S16	-0.21036	0.5803
scaffold1.g325	*RP-LP0*	large subunit ribosomal protein LP0	-0.42	0.74251
scaffold1.g328	*RP-S18e*	small subunit ribosomal protein S18e	-0.59627	0.81734
scaffold1.g329	*RP-L4*	large subunit ribosomal protein L4	-0.17934	0.39452
scaffold1.g348	*RP-S28e*	small subunit ribosomal protein S28e	0.01747	0.42167
scaffold1.g387	*RP-L7*	large subunit ribosomal protein L7/L12	-0.12353	0.71896
scaffold1.g439	*RP-S7*	small subunit ribosomal protein S7	-0.042838	0.96265
scaffold1.g441	*RP-S14*	small subunit ribosomal protein S14	0.21043	-0.22614
scaffold1.g457	*RP-S28e*	small subunit ribosomal protein S28e	-1.2754	0.039817
scaffold1.g578	*RP-L12e*	large subunit ribosomal protein L12e	-0.22204	0.6501
scaffold1.g633	*RP-L31e*	large subunit ribosomal protein L31e	-1.1367	0.92026
scaffold1.g672	*RP-S15Ae*	small subunit ribosomal protein S15Ae	-0.04582	0.5153
scaffold1.g735	*RP-L10*	large subunit ribosomal protein L10	0.19564	0.77739
scaffold1.g880	*RP-L35Ae*	large subunit ribosomal protein L35Ae	-0.67796	0.51413
scaffold1.g894	*RP-S8e*	small subunit ribosomal protein S8e	-0.55188	0.82744

scaffold1.g991	*RP-S18*	small subunit ribosomal protein S18	0.28513	0.89643
scaffold2.g180	*RP-L38e*	large subunit ribosomal protein L38e	-0.62691	0.67208
scaffold2.g238	*RP-S7e*	small subunit ribosomal protein S7e	-0.94132	0.79771
scaffold2.g245	*RP-S2e*	small subunit ribosomal protein S2e	-0.47666	0.90009
scaffold2.g310	*RP-S20e*	small subunit ribosomal protein S20e	-0.75425	0.80808
scaffold2.g364	*RP-L27Ae*	large subunit ribosomal protein L27Ae	-0.48183	0.55849
scaffold2.g374	*RP-S3e*	small subunit ribosomal protein S3e	-0.46812	0.90185
scaffold2.g39	*RP-S12e*	small subunit ribosomal protein S12e	-0.32495	0.6085
scaffold2.g72	*RP-L7Ae*	large subunit ribosomal protein L7Ae	-1.014	1.0525
scaffold2.g735	*RP-L17e*	large subunit ribosomal protein L17e	-0.60113	0.79548
scaffold2.g784	*RP-S10*	small subunit ribosomal protein S10	0.035029	0.71047
scaffold2.g827	*RP-S17*	small subunit ribosomal protein S17	0.13352	0.40216
scaffold3.g299	*RP-S4e*	small subunit ribosomal protein S4e	-0.90192	-0.041967
scaffold3.g302	*RP-L23*	large subunit ribosomal protein L23	-0.2813	1.2515
scaffold3.g304	*RP-L13*	large subunit ribosomal protein L13	0.53869	0.39814
scaffold3.g336	*RP-L30e*	large subunit ribosomal protein L30e	0.3755	1.1107
scaffold3.g337	*RP-L24e*	large subunit ribosomal protein L24e	-0.41198	0.63939
scaffold3.g363	*RP-S9*	small subunit ribosomal protein S9	-0.013316	0.87734
scaffold3.g376	*RP-L27e*	large subunit ribosomal protein L27e	-1.1358	0.5014
scaffold3.g461	*RP-L9e*	large subunit ribosomal protein L9e	-0.73167	0.63818
scaffold3.g489	*RP-S11*	small subunit ribosomal protein S11	0.2306	0.65684
scaffold3.g490	*RP-L23Ae*	large subunit ribosomal protein L23Ae	-0.85841	0.54796

scaffold3.g518	*RP-S25e*	small subunit ribosomal protein S25e	0.32977	0.76523
scaffold3.g535	*RP-S6e*	small subunit ribosomal protein S6e	-0.96197	0.8117
scaffold3.g541	*RP-L6*	large subunit ribosomal protein L6	0.26269	0.77371
scaffold3.g542	*RP-L3*	large subunit ribosomal protein L3	-0.026458	0.94767
scaffold3.g557	*RP-L4e*	large subunit ribosomal protein L4e	-0.033575	0.97945
scaffold3.g563	*RP-L3e*	large subunit ribosomal protein L3e	-0.3115	0.94847
scaffold3.g635	*RP-L28*	large subunit ribosomal protein L28	-0.14283	0.74467
scaffold3.g718	*RP-SAe*	small subunit ribosomal protein SAe	-1.2589	0.81702
scaffold3.g730	*RP-S5*	small subunit ribosomal protein S5	-0.33487	0.66139
scaffold3.g746	*RP-L29e*	large subunit ribosomal protein L29e	-0.44614	0.30534
scaffold4.g196	*RP-S27e*	small subunit ribosomal protein S27e	-1.3028	1.4053
scaffold4.g316	*RP-L13Ae*	large subunit ribosomal protein L13Ae	-0.39485	1.0678
scaffold4.g332	*RP-L14e*	large subunit ribosomal protein L14e	-0.78234	0.64686
scaffold4.g379	*RP-L21e*	large subunit ribosomal protein L21e	-0.84229	0.55893
scaffold4.g389	*RP-S26e*	small subunit ribosomal protein S26e	0.14124	0.72452
scaffold4.g40	*RP-L37e*	large subunit ribosomal protein L37e	-0.49884	0.77851
scaffold4.g490	*RP-L15e*	large subunit ribosomal protein L15e	-0.47862	0.89772
scaffold4.g499	*RP-L24*	large subunit ribosomal protein L24	-0.25357	0.55609
scaffold4.g85	*RP-S6*	small subunit ribosomal protein S6	-1.1212	0.32068
scaffold5.g24	*RP-L7e*	large subunit ribosomal protein L7e	-1.0022	1.0006
scaffold5.g362	*RP-LP1*	large subunit ribosomal protein LP1	0.042406	0.72199
scaffold5.g363	*RP-L13e*	large subunit ribosomal protein L13e	-0.57342	0.83957

scaffold5.g390	*RP-S3Ae*	small subunit ribosomal protein S3Ae	-0.58285	0.84186
scaffold5.g62	*RP-L23e*	large subunit ribosomal protein L23e	-0.43121	1.0229
scaffold5.g83	*RP-S2*	small subunit ribosomal protein S2	0.1767	0.34224
scaffold6.g107	*RP-L19e*	large subunit ribosomal protein L19e	-0.28065	0.62558
scaffold6.g116	*RP-S15e*	small subunit ribosomal protein S15e	-0.8192	0.51867
scaffold6.g117	*RP-LP2*	large subunit ribosomal protein LP2	-0.070676	0.81575
scaffold6.g190	*RP-L27*	large subunit ribosomal protein L27	0.23768	-0.11965
scaffold6.g245	*RP-L32*	large subunit ribosomal protein L32	-0.32888	0.65135
scaffold6.g261	*RP-S19e*	small subunit ribosomal protein S19e	-0.54546	1.1633
scaffold6.g300	*RP-S12*	small subunit ribosomal protein S12	-0.2843	0.5527
scaffold6.g385	*RP-L5e*	large subunit ribosomal protein L5e	-0.68951	0.98893
scaffold6.g42	*RP-S15Ae*	small subunit ribosomal protein S15Ae	-1.5115	0.65172
scaffold6.g459	*RP-L11e*	large subunit ribosomal protein L11e	-0.6055	0.68894
scaffold6.g78	*RP-S17e*	small subunit ribosomal protein S17e	-1.0515	0.29422
scaffold7.g151	*RP-S23e*	small subunit ribosomal protein S23e	-0.46036	0.85965
scaffold7.g215	*RP-S5e*	small subunit ribosomal protein S5e	-0.83263	1.0721
scaffold7.g275	*RP-L15*	large subunit ribosomal protein L15	-0.083718	0.58354
scaffold7.g321	*RP-S15*	small subunit ribosomal protein S15	-0.02548	0.3484
scaffold8.g96	*RP-L10Ae*	large subunit ribosomal protein L10Ae	-0.11275	0.75388

Table S2. Differentially expressed genes involved in the fructose and mannose metabolism (KEGG: pgu00051) in the Cu-600 and Cu-1000 groups.

Locus	Gene symbol	Gene description	log2Cu600/Cu0	log2Cu1000/Cu0
scaffold1.g1023	*PFKFB4*	6-phosphofructo-2-kinase	0.39285	0.57897
scaffold1.g1035	*PMM*	phosphomannomutase	-0.79074	0.39221
scaffold1.g191	*DAK,*	triose/dihydroxyacetone kinase	0.23508	-0.54501
scaffold1.g860	*HK;*	hexokinase	0.90646	-1.9597
scaffold2.g144	*MPI*	mannose-6-phosphate isomerase	-0.4989	-0.77222
scaffold2.g316	*PFKFB2;*	6-phosphofructo-2-kinase	0.33245	0.25563
scaffold2.g673	*PFK*	6-phosphofructo-2-kinase	0.24187	-0.95661
scaffold2.g789	*TPI*	triosephosphate isomerase	0.51985	-1.7765
scaffold2.g957	*SORD*	L-iditol 2-dehydrogenase	2.1307	2.2314
scaffold3.g144	*pfkA*	6-phosphofructokinase 1	-0.0078584	-2.2875
scaffold3.g509	*HK*	hexokinase	0.68586	-1.5064
scaffold3.g759	*PFK*	6-phosphofructo-2-kinase	0.72418	-1.1493
scaffold4.g288	*LRA3*	L-rhamnonate dehydratase	0.44133	-0.086821
scaffold4.g290	*LRA4*	2-keto-3-deoxy-L-rhamnonate aldolase	0.55708	-0.3044
scaffold4.g291	*LRA1*	L-rhamnose 1-dehydrogenase	1.8363	2.2911
scaffold4.g508	*FBA*	fructose-bisphosphate aldolase, class II	-1.3369	-0.26871
scaffold5.g418	*PFK*	6-phosphofructokinase 1	0.24893	-2.2128
scaffold6.g475	*FBP*	fructose-1,6-bisphosphatase I	0.63722	-0.44332
scaffold6.g477	*GMPP*	mannose-1-phosphate guanylyltransferase	-0.46025	0.32246
scaffold7.g171	*SOU1*	sorbose reductase	1.4105	0.33364
scaffold7.g379	*FBA*	fructose-bisphosphate aldolase, class II	0.78289	-1.9305

Table S3. DEGs involved in the Biosynthesis of amino acids metabolism (KEGG: pgu01230) in Cu-600 and Cu-1000 groups.

Locus	Gene symbol	Gene description	log2Cu600/Cu0	log2Cu1000/C0
scaffold1.g1012	*LEU1*	3-isopropylmalate dehydratase	-0.21034	1.8219
scaffold1.g160	*E2.5.1.54*	3-deoxy-7-phosphoheptulonate synthase	-0.46514	1.4286
scaffold1.g163	*ARG2*	amino-acid N-acetyltransferase	-0.43538	-0.29455
scaffold1.g174	*HIS7*	imidazole glycerol-phosphate synthase	0.075367	0.96067
scaffold1.g202	*thrC*	threonine synthase	-0.13647	0.33893
scaffold1.g217	*argJ*	glutamate N-acetyltransferase	-0.059356	0.61225
scaffold1.g48	*argG*	argininosuccinate synthase	-0.15664	-0.54279
scaffold1.g482	*E2.5.1.54*	3-deoxy-7-phosphoheptulonate synthase	-0.078326	-0.69212
scaffold1.g631	*PK*	pyruvate kinase	0.046357	-1.5655
scaffold1.g84	*ACO*	aconitate hydratase	0.43406	0.084877
scaffold1.g847	*rpe*	ribulose-phosphate 3-epimerase	0.71037	-0.36746
scaffold1.g890	*CTH*	cystathionine gamma-lyase	1.1937	-0.10401
scaffold1.g905	*hisB*	imidazoleglycerol-phosphate dehydratase	0.41147	0.72112
scaffold1.g917	*CBS*	cystathionine beta-synthase	0.75306	0.86278
scaffold2.g117	*GPT*	alanine transaminase	0.096432	-0.8223
scaffold2.g169	*metE*	5-methyltetrahydropteroyltriglutamate--homocysteine methyltransferase	-0.94228	-0.38117
scaffold2.g194	*metC*	cysteine-S-conjugate beta-lyase	0.4487	0.53253
scaffold2.g239	*rpiA*	ribose 5-phosphate isomerase A	0.22501	0.019705
scaffold2.g24	*E4.3.1.19*	threonine dehydratase	0.63238	-0.056544
scaffold2.g401	*ARG56*	N-acetyl-gamma-glutamyl-phosphate reductas	-0.10599	-0.38903
scaffold2.g42	*proA*	glutamate-5-semialdehyde dehydrogenase	0.19089	0.47551
scaffold2.g442	*leuA*	2-isopropylmalate synthase	0.58726	0.8316
scaffold2.g470	*TRP3*	anthranilate synthase	0.04413	0.67527
scaffold2.g508	*serA*	D-3-phosphoglycerate dehydrogenase	0.64913	0.6658
scaffold2.g543	*trpE*	anthranilate synthase component I	-0.14732	0.46408
scaffold2.g582	*E2.2.1.6S*	acetolactate synthase I	-0.46338	0.40052
scaffold2.g676	*ilvC*	ketol-acid reductoisomerase	-0.74262	2.4196

scaffold2.g698	*HIS4*	phosphoribosyl-ATP pyrophosphohydrolase	-0.78548	0.37099
scaffold2.g774	*LYS4*	homoaconitate hydratase	0.047819	-0.35462
scaffold2.g777	*argE*	acetylornithine deacetylase	-0.27643	-0.78004
scaffold2.g811	*proC*	pyrroline-5-carboxylate reductase	0.19977	-0.26595
scaffold2.g902	*E2.2.1.1*	transketolase	0.51032	-0.50801
scaffold3.g109	*E3.5.3.1*	arginase	1.7119	3.9894
scaffold3.g161	*serB*	phosphoserine phosphatase	-0.18513	-0.10105
scaffold3.g19	*cysK*	cysteine synthase	0.69137	0.50655
scaffold3.g237	*IDH3*	isocitrate dehydrogenase (NAD+)	0.33129	0.27821
scaffold3.g330	*IDH1*	isocitrate dehydrogenase	0.96882	0.69492
scaffold3.g404	*E2.5.1.54*	3-deoxy-7-phosphoheptulonate synthase	-0.2126	0.95574
scaffold3.g507	*glyA*	glycine hydroxymethyltransferase	0.91355	1.8938
scaffold3.g640	*E2.6.1.42*	branched-chain amino acid aminotransferase	-0.16582	0.19294
scaffold3.g693	*TYR1*	prephenate dehydrogenase (NADP+)	-0.035098	0.09986
scaffold4.g137	*thrB*	homoserine kinase	0.10105	0.28665
scaffold4.g192	*aroC*	chorismate synthase	-0.18643	0.20794
scaffold4.g317	*leuA*	2-isopropylmalate synthase	-0.076178	0.98644
scaffold4.g322	*trpF*	phosphoribosylanthranilate isomerase	0.11081	0.18856
scaffold4.g351	*IDH1*	isocitrate dehydrogenase	0.059291	-0.28133
scaffold4.g362	*ARO1*	pentafunctional AROM polypeptide	-0.43223	0.22232
scaffold4.g440	*PC*	pyruvate carboxylase	0.54462	0.58972
scaffold4.g485	*PGK*	phosphoglycerate kinase	0.83232	-2.0599
scaffold4.g540	*LYS12*	homoisocitrate dehydrogenase	0.68954	0.33979
scaffold4.g578	*GPT*	alanine transaminase	0.22722	0.70448
scaffold5.g128	*PGAM*	2，3-bisphosphoglycerate-dependent phosphoglycerate mutase	0.43599	-2.204
scaffold5.g285	*TRP*	tryptophan synthase	0.40327	0.32658
scaffold5.g304	*PGAM*	2，3-bisphosphoglycerate-dependent phosphoglycerate mutase	0.60775	-1.4132
scaffold5.g32	*dapA*	4-hydroxy-tetrahydrodipicolinate synthase	1.2451	0.50491
scaffold5.g336	*metX*	homoserine O-acetyltransferase	0.13296	0.50667
scaffold5.g344	*LYS2*	L-2-aminoadipate reductase	-0.107	-0.029513

scaffold5.g385	*metB*	cystathionine gamma-synthase	0.55546	0.58885
scaffold6.g289	*cysK*	cysteine synthase	-0.37825	0.24912
scaffold6.g33	*GLT1*	glutamate synthase (NADH)	-0.49891	1.5843
scaffold6.g467	*hom*	homoserine dehydrogenase	0.70664	0.60267
scaffold7.g234	*hisA*	phosphoribosylformimino-5-aminoimidazole carboxamide ribotide isomerase	0.4801	0.5027
scaffold7.g262	*LYS1*	saccharopine dehydrogenase (NAD+)	-0.55445	0.25307
scaffold7.g324	*GOT2*	aspartate aminotransferase	-0.33438	-0.73478
scaffold7.g55	*glyA*	glycine hydroxymethyltransferase	0.17752	-0.068497
scaffold8.g149	*metX*	homoserine O-acetyltransferase	-0.46655	0.79809

Table S4. Differentially expressed genes involved in the Glutathione metabolism (KEGG: map00480) in the Cu-600 and Cu-1000 groups.

Locus	Gene symbol	Gene description	log2Cu600/Cu0	log2Cu1000/Cu0
scaffold1.g532	*GST*	glutathione S-transferase	0.51004	-3.9293
scaffold1.g533	*GST*	glutathione S-transferase	0.115	-1.84
scaffold1.g534	*GST*	glutathione S-transferase	-0.91645	0.40867
scaffold1.g892	*gshB*	glutathione synthase	-0.23349	0.57351
scaffold2.g557	*OPLAH*	5-oxoprolinase (ATP-hydrolysing)	0.61328	-1.0049
scaffold2.g678	*gpx*	glutathione peroxidase	0.66605	-0.69912
scaffold2.g753	*GST*	glutathione S-transferase	0.19355	0.36841
scaffold3.g117	*DUG1*	Cys-Gly metallodipeptidase DUG1	0.031612	0.45419
scaffold3.g178	*RRM2*	ribonucleoside-diphosphate reductase subunit M2	-1.7226	-1.0967
scaffold3.g180	*OPLAH*	5-oxoprolinase (ATP-hydrolysing)	0.1347	-0.60506
scaffold3.g229	*RRM1*	ribonucleoside-diphosphate reductase subunit M1	-2.3178	0.26808
scaffold3.g330	*IDH1*	isocitrate dehydrogenase	0.96882	0.69492
scaffold3.g56	*GCLC*	glutamate--cysteine ligase catalytic subunit	-0.039606	0.95941
scaffold4.g12	*GST*	glutathione S-transferase	0.936	1.8256
scaffold4.g351	*IDH1*	isocitrate dehydrogenase	0.059291	-0.28133
scaffold4.g412	*G6PD*	glucose-6-phosphate 1-dehydrogenase	0.51536	0.21882
scaffold4.g64	*GSR*	glutathione reductase	0.71881	0.88797
scaffold5.g107	*speE*	spermidine synthase	-0.066464	-0.17067
scaffold5.g379	*ggt*	gamma-glutamyltranspeptidase	0.8991	0.48682
scaffold5.g397	*E4.1.1.17*	ornithine decarboxylase	-0.43337	-0.018438
scaffold5.g80	*PGD*	6-phosphogluconate	0.64837	-0.8472

		dehydrogenase		
scaffold6.g308	*gpx*	glutathione peroxidase	0.55097	-1.4138
scaffold6.g361	*RRM1*	ribonucleoside-diphosphate reductase subunit M1	0.75962	1.5078
scaffold7.g414	*OPLAH*	5-oxoprolinase (ATP-hydrolysing)	0.101	0.16072
scaffold8.g106	*OPLAH*	5-oxoprolinase (ATP-hydrolysing)	0.30148	-0.04878
scaffold8.g9	*OPLAH*	5-oxoprolinase (ATP-hydrolysing)]	0.76918	0.18892
scaffold9.g41	*ggt*	gamma-glutamyl transpeptidase	0.49458	-1.7653
scaffold9.g97	*pxpA*	5-oxoprolinase (ATP-hydrolysing) subunit A	1.4239	3.0649

Additional file 2. Analysis of GXDK6 fructose and mannose metabolism pathway under copper stress

Fig. S1. Analysis of GXDK6 fructose and mannose metabolism pathway under copper stress.The pathway of Fructose and D-mannose metabolism in GXDK6. A: hexokinase (encoded by *HK* which was downregulated in Cu-1000 group); B: fructose-bisphosphate aldolase (encoded by *FBA* which was most downregulated in Cu-600 group; C: 6-phosphofructokinase 1(encoded by *pfkA* which was most downregulated in Cu-1000 group); D: L-iditol 2-dehydrogenase (encoded by *SORD* which was most upregulated in Cu-600 group); E: sorbose reductase (encoded by *SOU1* which was upregulated in Cu-600 group).

Fig. S1.

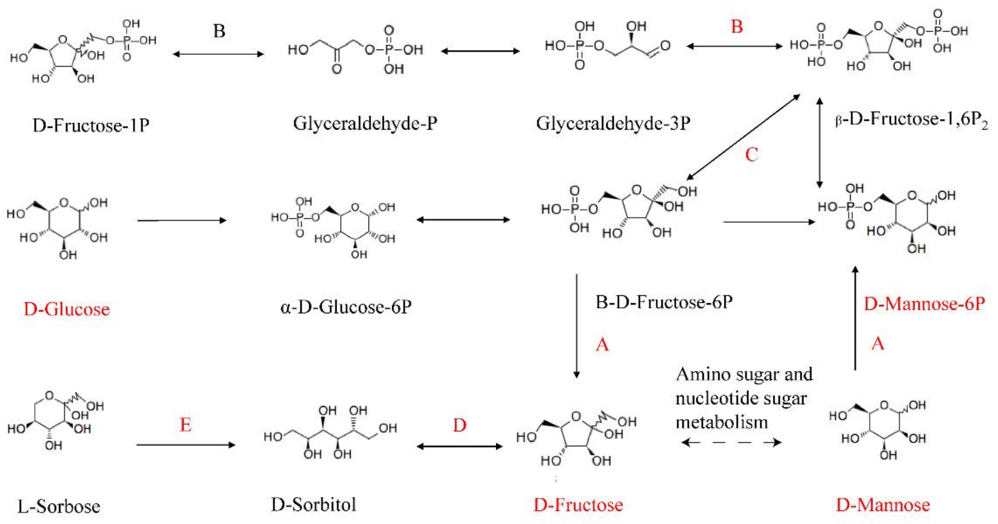

A B C D E

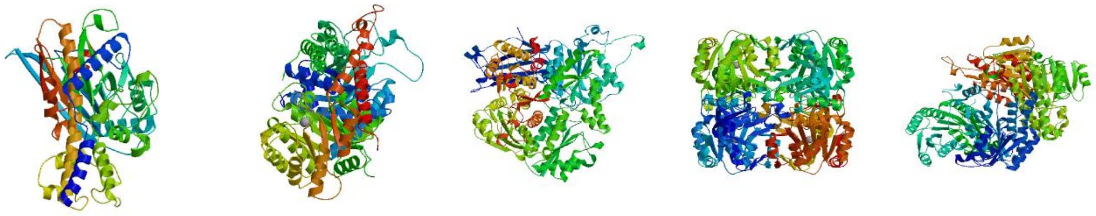

Additional file 3. Analysis of the effect of exogenous addition of D-mannose on the growth of GXDK6 under copper stress

Fig. S2. The effect of exogenous addition of D-mannose on the growth of GXDK6 under copper stress. A: In Cu-600 group, the effect of exogenous addition of D-mannose on the growth of GXDK6, the addition of 0-0.3% of exogenous D-mannose can promote the growth of GXDK6 under copper ions stress. B: in the Cu-1000 group, the effect of exogenous addition of D-mannose on the growth of GXDK6. The addition of 0-0.6% of exogenous D-mannose can promote the growth of GXDK6 under copper ions stress.

Fig. S2.

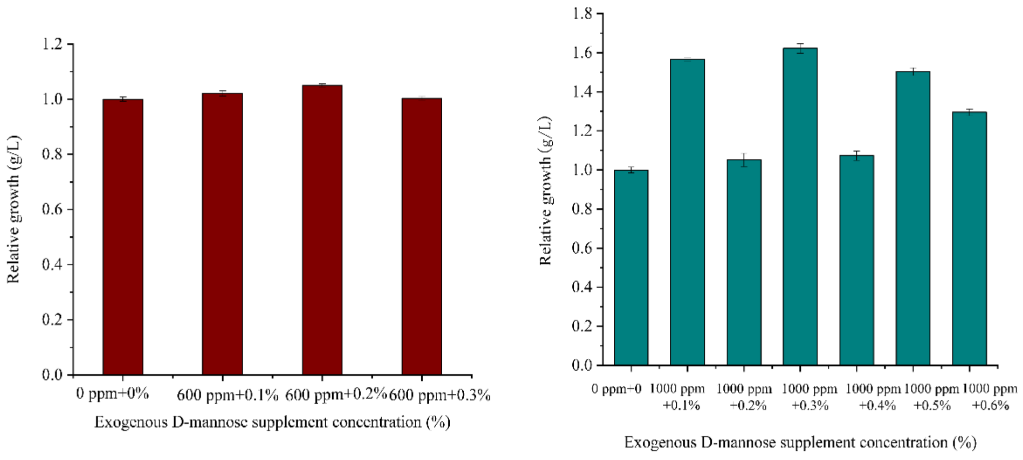

A B

Additional file 4. Key copper tolerance gene expression verification

Fig. S3. Validation of key genes related to copper tolerance by RT-qPCR.SYBR Green RT-qPCR was used to validate the expression of genes related to copper ions tolerance selected by RNA-Seq the expression of *CAT*, *GST*, and *GLR1* in Cu-1000 group was up-regulated by 2.67, 1.41, and 2.96 times, respectively, and there was no significant difference in *SOD2* expression, which was consistent with the transcriptome data, indicating high reliability of the RNA-seq analysis.

Fig. S3.

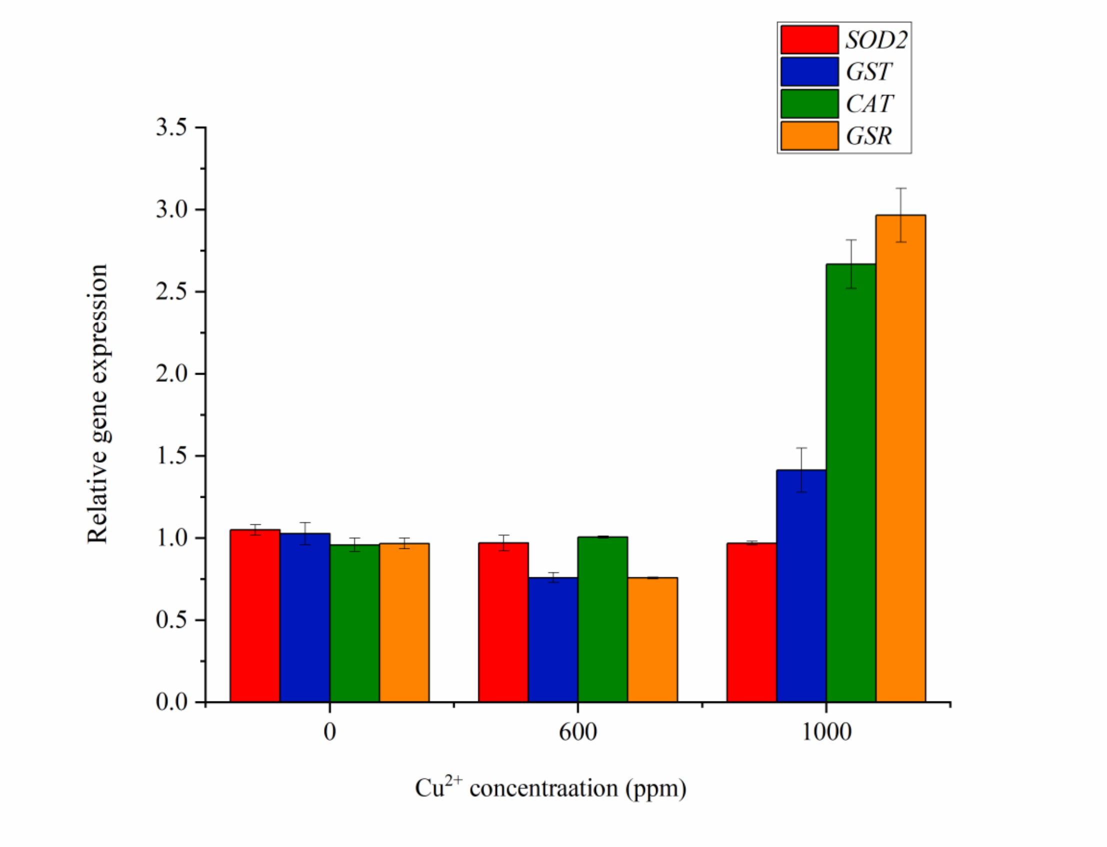

Supplement: Supplementary file 1 [file Presentation_1.pdf]
